# Is the radiographic subsidence of stand-alone cages associated with adverse clinical outcomes after cervical spine fusion? An observational cohort study with 2-year follow-up outcome scoring

**DOI:** 10.1186/s13037-014-0043-4

**Published:** 2014-11-07

**Authors:** Dirk Zajonz, Anne-Catherine Franke, Nicolas von der Höh, Anna Voelker, Michael Moche, Jens Gulow, Christoph-Eckhard Heyde

**Affiliations:** Department of Orthopaedic Surgery, Traumatology and Plastic Surgery, University Hospital Leipzig, Liebigstrasse 20, 04103 Leipzig, Germany; Department of Diagnostic and Interventional Radiology, University Hospital Leipzig, Liebigstrasse 20, 04103 Leipzig, Germany

**Keywords:** Cervical cage subsidence, ACDF, Stand-alone cervical cages, Cervical spine

## Abstract

**Background:**

The stand-alone treatment of degenerative cervical spine pathologies is a proven method in clinical practice. However, its impact on subsidence, the resulting changes to the profile of the cervical spine and the possible influence of clinical results compared to treatment with additive plate osteosynthesis remain under discussion until present.

**Methods:**

This study was designed as a retrospective observational cohort study to test the hypothesis that radiographic subsidence of cervical cages is not associated with adverse clinical outcomes. 33 cervical segments were treated surgically by ACDF with stand-alone cage in 17 patients (11 female, 6 male), mean age 56 years (33–82 years), and re-examined after eight and twenty-six months (mean) by means of radiology and score assessment (Medical Outcomes Study Short Form (MOS-SF 36), Oswestry Neck Disability Index (ONDI), painDETECT questionnaire and the visual analogue scale (VAS)).

**Results:**

Subsidence was observed in 50.5% of segments (18/33) and 70.6% of patients (12/17). 36.3% of cases of subsidence (12/33) were observed after eight months during mean time of follow-up 1. After 26 months during mean time of follow-up 2, full radiographic fusion was seen in 100%. MOS-SF 36, ONDI and VAS did not show any significant difference between cases with and without subsidence in the two-sample t-test. Only in one type of scoring (painDETECT questionnaire) did a statistically significant difference in t-Test emerge between the two groups (p = 0.03; α = 0.05). However, preoperative painDETECT score differ significantly between patients with subsidence (13.3 falling to 12.6) and patients without subsidence (7.8 dropped to 6.3).

**Conclusions:**

The radiological findings indicated 100% healing after stand-alone treatment with ACDF. Subsidence occurred in 50% of the segments treated. No impact on the clinical results was detected in the medium-term study period.

## Background

Neck pain is one of the most common reasons for visiting the doctor in Western countries with a worldwide point prevalence (age 15–74 years) of 7.6% (5.9–38.7%) and a lifetime prevalence (age 18–84 years) of 48.5% (14.2–71%) [1–5]. It therefore constitutes a significant economic factor in healthcare. In fact in Germany alone, in 2002 the treatment of spinal conditions accounted for 3.2% (€7.2 billion) of gross healthcare spending [6].

The symptoms are increasingly caused by degenerative processes with rising age. For example, degenerative changes to the cervical spine, especially degenerative disc disease with neural foramen and spinal canal stenosis, occur in almost 95% of over-seventies [3,4]. Once conservative options have been exhausted as well as in cases with distinct neurologic symptoms surgery is the treatment of choice. The standard method is ventral decompression and spinal fusion [7]. For many years, the main form of fusion was the insertion of an autologous bone graft of the same height [8–10]. In the 1960s, fusion by bone graft was supplemented by ventral plates to improve the stability, to avoid los of height as well as consecutive kyphosis and thus to optimize healing [11,12]. Additive plating shortened the postoperative immobilization period until bony consolidation and reduced pseudarthrosis rate [11,13,14]. However, the long-term results revealed complications due to additional soft tissue compression caused by the plate and the need in some cases for more extensive surgery. In a retrospective analysis of 1015 patients treated with ACDF, including 95.7% (971) provided with a ventral plate, Fountas et al. 2007 reported dysphagia in 8.1% (82), paralysis of the recurrent laryngeal nerve 2.9% (29), 1 case of Horner’s syndrome, and 3 cases of oesophageal perforation, including one with a fatal outcome [14,15].

Since the early 1990s interbody implants have been increasingly used to avoid loss of height, kyphosis and to reduce pseudarthrosis rate. These rigid cages take the form of a hollow body. The additional insertion of bone material or bone-inductive substances means that secondary fusion is also possible without additive ventral plate osteosynthesis [16]. Over time, various materials have been used ranging from metal alloys (titanium, etc.) and synthetic materials (PMMA, PEEK, etc.) to biomaterials [8,10,17,18].

A meta-analysis published by Schröder and colleagues in 2002 followed up approximately 8600 cervical discectomies and fusions. No surgical procedures or fusion materials were found to have clear advantages [19]. However, increasing cage subsidence in the endplates of the adjacent vertebral bodies following stand-alone treatment was striking. Although there is disagreement whether this subsidence affects stability or the outcome, in recent studies no such effect has been ascertained [20–24]. The aim of our study was to examine whether clinical outcome is impacted by postoperative cage subsidence and the resulting change in profile.

## Materials and methods

This study was designed as a retrospective observational cohort study to test the hypothesis that radiographic subsidence of cervical cages is not associated with adverse clinical outcomes.

From January 13, 2010 until November 3, 2011, 33 stand-alone cages were implanted in 17 patients with degenerative cervical spine disorders by means of ACDF. The mean age of the patients was 56 years (33–82 years). There were 11 female patients (65%; mean age 55 years, 33–82) and 6 males (35%; mean age 59 years, 47–72). Fusion was monosegmental in 5 patients, bisegmental in 8 patients, and trisegmental in 4 patients.

**Figure 1:**
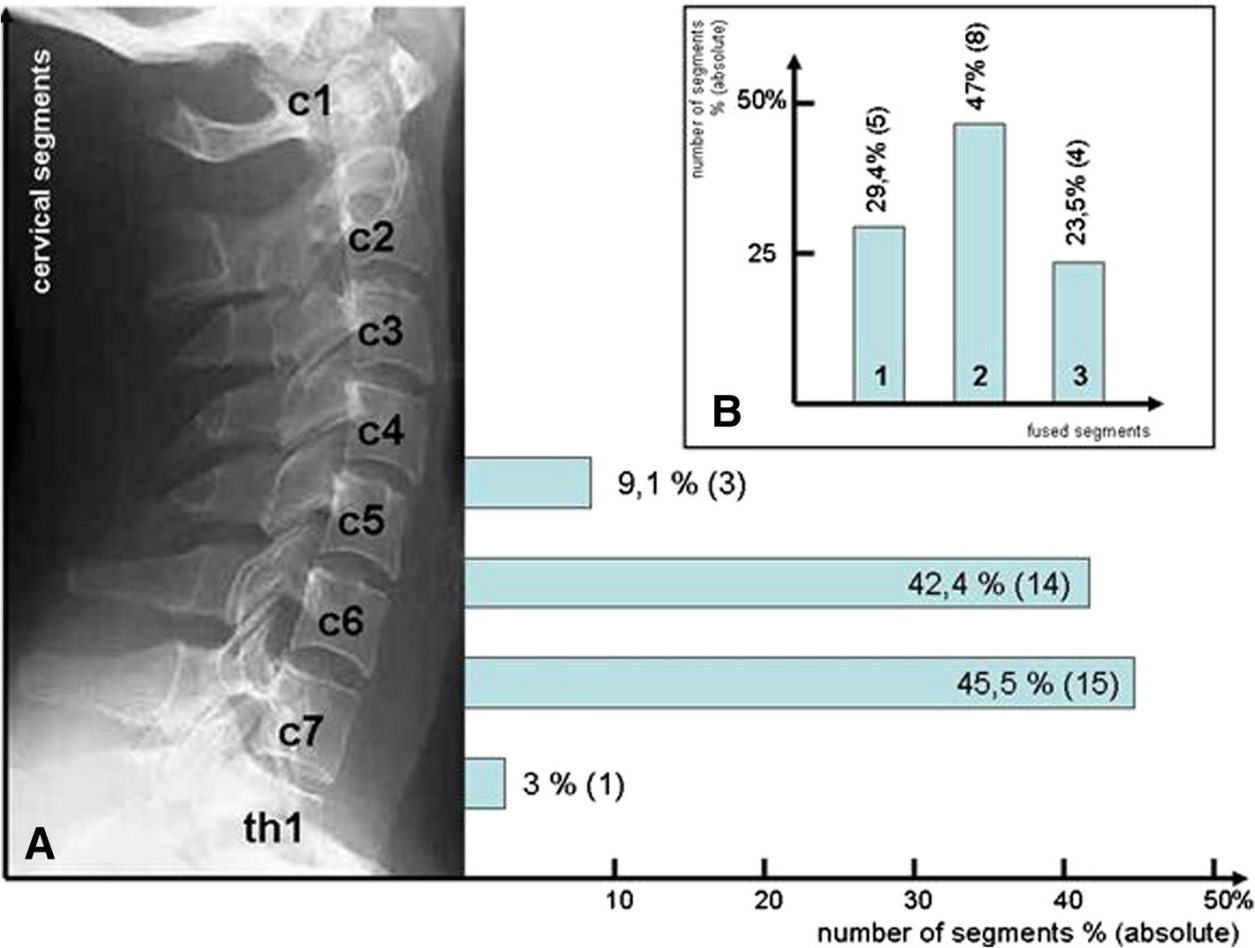
**Graph showing A percentage and absolute segment distribution by level (left) and B percentage and absolute distribution by monosegmental (1), bisegmental (2) and trisegmental (3) fusion (top right).**

Of the total of 33 cages implanted, 3 (9.1%) were inserted into segment C 4/5, 14 (42.4%) into C 5/6, 15 (45.5%) into C6/7, and 1 (3%) into C7/TH1 (Figure 1). Only patients with degenerative disorders of the cervical spine treated solely by means of ventral fusion involving cage interposition after decompression were included in the study.

Surgery was performed under general anaesthesia after standardized preparation and individual planning using the Smith-Robinson procedure aided by a microscope [25]. After decompression of the corresponding intervertebral disc space, the removal of dorsal or dorsolateral spondylophytes and the resection of the posterior longitudinal ligament nerve roots were examined and if necessary exposed. The superior and inferior endplates were carefully debrided. Cage implantation was carried out following size check under clinical and radiological control.

**Figure 2:**
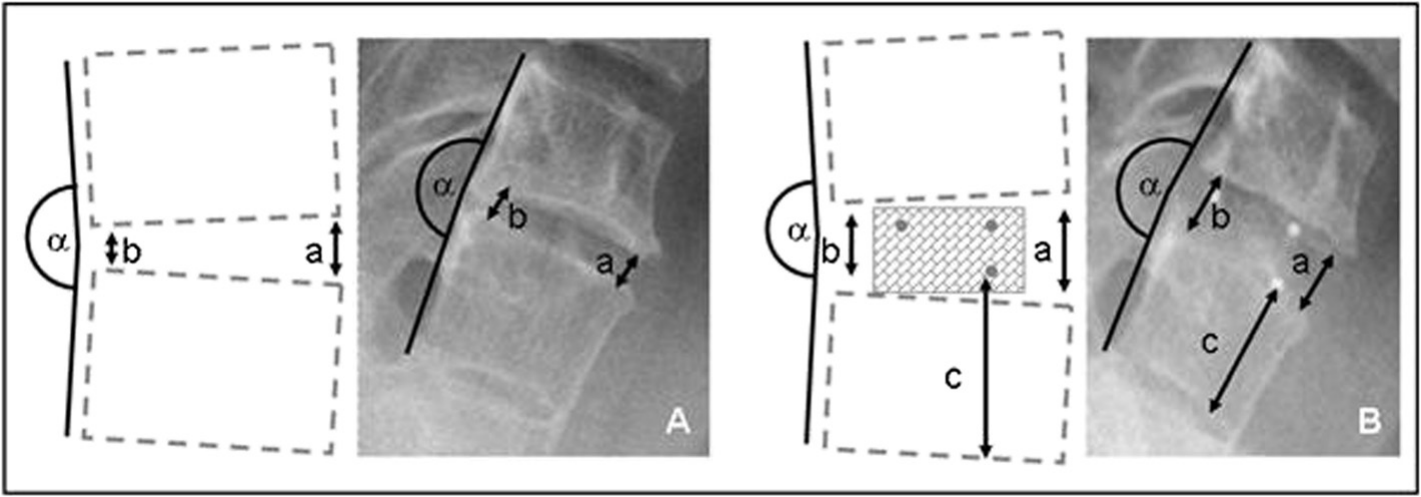
**Diagram showing the measurements in the lateral X-rays taken A preoperatively and B postoperatively: a ventral intervertebral space between two vertebrae along the anterior edge of the vertebral bodies, b dorsal intervertebral space between two vertebrae along the posterior edge of the vertebral bodies, α angle between the posterior edges of two adjacent vertebral bodies, c distance between the X-ray marker and the inferior endplate as a measure of subsidence.**

The cervical CFRP (carbon fibre reinforced PEEK) I/F Cage® system (DePuy Synthes Spine Inc, Raynham, MA, USA) was used. The basic matrix consists of a combination of PEEK (polyether ether ketone) and carbon fibre re-embedded [26]. X-ray markers are embedded in the cage for visualization (Figure 2). The sizes used were standard (15 × 12 mm, breadth and depth) and large (18 × 14 mm) with a lordotic angle of 7°. The height of the cages was planned preoperatively and determined intraoperatively by measuring the intervertebral space (4–8 mm in 2 mm increments). Implantation was carried out by means of appropriate instruments developed by the manufacturer for one-handed spinal fusion without additional support elements.

Anterior-posterior (AP) and lateral X-rays of the cervical spine were produced within the first 1–8 days after surgery (mean: 2.2 days) and again after 6 weeks. The first mean time of follow-up (mean time of follow-up 1) in the study was carried out after a mean of 8 months (6–13 months) and mean time of follow-up 2 after a mean of 26 months (23–37 months). Radiological measurements were carried out on the basis of standardized digitized conventional X-rays in the lateral view. The measurements were taken on the user interface using the integrated SIENET MagicWeb software (Siemens Medical Solutions, Erlangen, Germany). Measurements were taken of the ventral intervertebral space (the distance between two adjacent vertebral bodies along their anterior edge; symbol a), the dorsal intervertebral space (the distance between two adjacent vertebral bodies along their posterior edge; symbol b) and the angle between the rear edges of two adjacent vertebral bodies as a measure of uprightness (symbol α) (Figure 2). In addition, the subsidence of the cage into the superior and inferior endplates was measured. In line with the current literature, subsidence was defined as a loss of height of at least 2 mm [21,27,28]. Smaller readings could not be validated due to the standard measurement error of the software. Subsidence was measured as the distance between the cage edges or the X-ray markers and the adjacent superior or inferior endplate (symbol c) compared to immediate postoperative measurement. A diagram of the measurements taken is contained in Figure 2.

In addition to clinical and radiological examination, the scores were evaluated before surgery and during follow-up. The scores used were the Oswestry Neck Disability Index (ONDI) as a gauge of everyday impairment [29–31], the painDETECT questionnaire to assess pain [32], the Medical Outcomes Study (MOS) 36-item short-form health survey (SF-36) to assess health status and quality of life [33], and the visual analogue scale (VAS) for the optical assessment of subjective pain intensity [34].

For statistical analysis, statistical significance was calculated using the two-sample t-test for two dependent samples. The level of significance was assumed to be 1% (α = 0.01).

## Results

Mean time of follow-up 1 was performed after a mean of 8 months (6–13 months); mean time of follow-up 2 was carried out after a mean of 26 months (23–37 months). The drop-out rate was 12% (2/17) for mean time of follow-up 1 and 23% (4/17) for mean time of follow-up 2.

**Figure 3:**
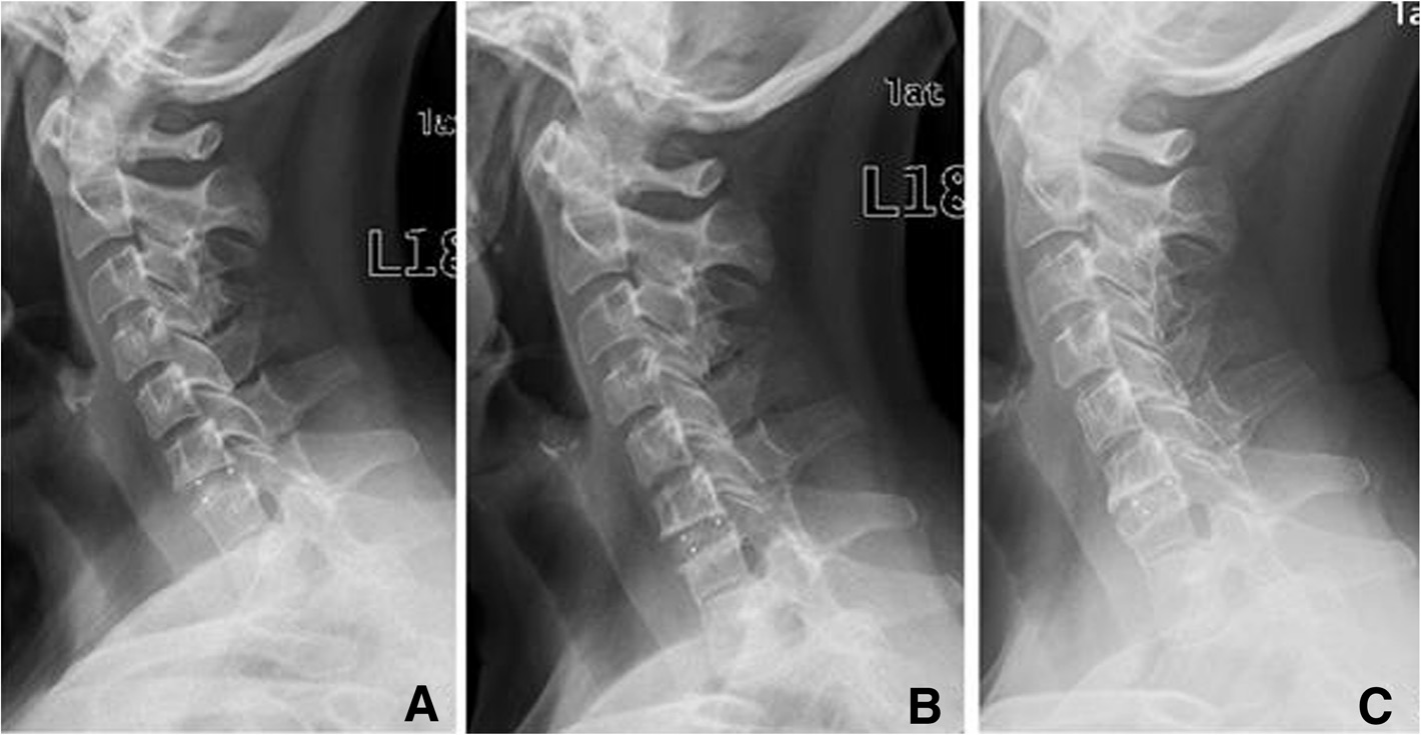
**Cervical spine (female, 59 years) with spinal stenosis and advanced disc degeneration in C6/7 and spontaneous block vertebra formation in C7/Th1 treated with a cervical cage in C6/7 – X-rays taken A immediately postoperatively, B 1 year after surgery with initial ventral subsidence of the cage into the superior endplate of C7, and C 2.5 years postoperatively with advanced ventral subsidence and osseous bridging.**

**Figure 4:**
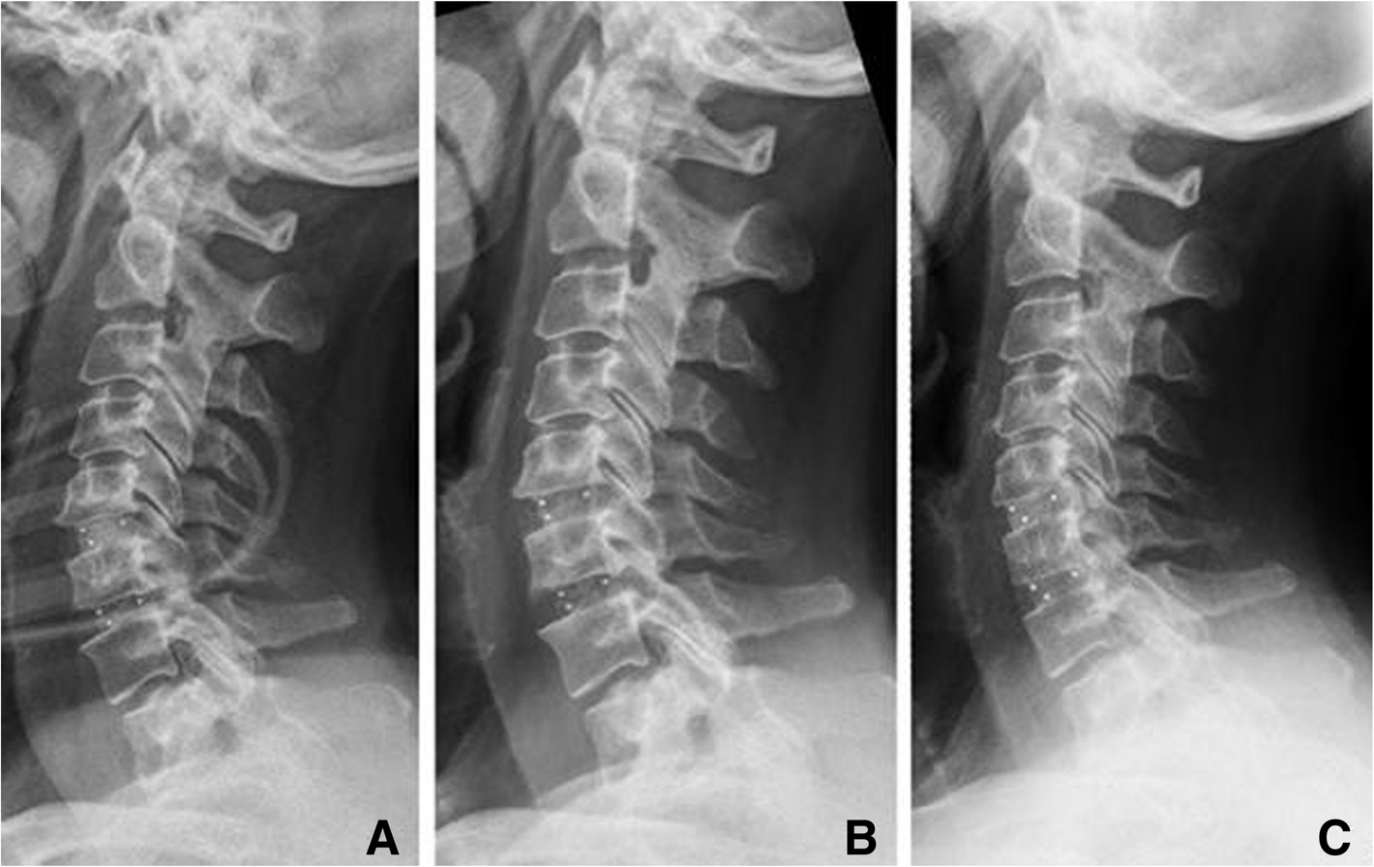
**Cervical spine (female, 66 years) with spinal stenosis in C5/6 and C6/7 and myelopathy treated by cervical cages in C5/6 and C6/7 – X-rays taken A immediately postoperatively, B 1 year after surgery without cage subsidence, and C 3 years postoperatively with osseointegration without subsidence.**

Of the total of 33 surgically treated segments, 18 (50.5%) indicated subsidence during the study. Subsidence was observed in 12 of the 33 segments (36.4%) in 11 patients during the first radiological follow-up (Figures 3 and 4). They comprised 12 cases of ventral subsidence (5 in the inferior endplate and 9 in the superior plate, including 2 in both endplates) and 7 of dorsal subsidence (3 in the inferior endplate and 5 in the superior endplate, including 1 in both endplates).

The mean ventral subsidence was 3.58 mm (2–7 mm) and the mean dorsal subsidence 2.4 mm (2–5 mm). In mean time of follow-up 2, 50.5% of the segments (18 out of 33) in 12 patients showed subsidence: 17 instances of ventral and 8 of dorsal subsidence; both ventral and dorsal subsidence were observed in 7 segments in 7 patients. The mean ventral subsidence was 4.18 mm (2–8 mm) and the mean dorsal subsidence was 2.75 mm (2–5 mm).

The mean preoperative kyphosis angle α between the dorsal edges of affected vertebral bodies was 176° (163–183°). Immediately postoperatively, the angle improved by about 4° to 172.5° (160–181°). By mean time of follow-up 1, the mean angle was 176.9° (157–190°), and 176.4° (156–196°) by mean time of follow-up 2. The changes to the individual measurements are shown in Table 1.

**Table 1:** **Development of radiological measurements**

	**Preoperative**	**Postoperative**	**FU1**	**FU2**
		**After mean 2.2 days (1–8 days)**	**After mean 8 months (6–13 months)**	**After mean 26 months (23–37 months)**
**α (kyphosis angle)**	176 (163–183)	172.5 (160–181)	176.9 (157–190)	176.4 (156–196)
**a (ventral intervertebral space) in mm**	3.6 (1.7–8.5)	7.5 (4.5–10.1)	6.2 (2.4–9)	5.6 (1.2–8.8)
**b (dorsal intervertebral space) in mm**	3.1 (1.2–8.5)	6.1 (3.9–7.7)	5.4 (3.3–7.4)	4.9 (0.7–7.6)
Subsided segments			36.3% (12 out of 33)	50.5% (18 out of 33)
Patients with subsidence			64.7% (11 out of 17)	70.6% (12 out of 17)

(α: kyphosis angle between two adjacent segments, a: ventral intervertebral space between two adjacent segments, b: dorsal intervertebral space between two adjacent segments) and subsidence by segment and patients at the individual survey times (preoperatively, postoperatively after 2.2 days (mean; 1–8 days), mean time of follow-up 1 after 8 months (mean; 6–13 months) and mean time of follow-up 2 after 26 months (mean; 23–37 months).

- The mean preoperative VAS of the total cohort was 5.9 (minimum 2 – maximum 8). In mean time of follow-up 1, the mean was 4.4 (1–9), and in mean time of follow-up 2 3.8 (2–8). Although the VAS indicated no significant improvement from before surgery to mean time of follow-up 1 (p = 0.09, α = 0.05) or between the two follow-ups, a significant improvement was noted between the preoperative VAS and mean time of follow-up 2 (p = 0.006, α = 0.05).
- On the painDETECT questionnaire, the total cohort scored 11.4 (mean; 4–21) preoperatively, which increased slightly in mean time of follow-up 1 to 11.8 (7–26) before dropping again in mean time of follow-up 2 to 10.9 (3–20). There were no significant improvements.
- The preoperative Oswestry Neck Disability Index (ONDI) indicating how patients’ neck pain affected their ability to manage everyday activities was 48.6% (mean; 8–90%) for the total cohort, which dropped in mean time of follow-up 1 to 36.8% (mean; 10–72%) and 36.4% (mean; 12–64%) in mean time of follow-up 2. However, the changes were not statistically significant.
- The mean preoperative Physical Component Summary (PCS; a measure of the physical quality of life) of the total cohort was 32 (20–46), rising to first 33 (17–50) in mean time of follow-up 1 and 37 (24–49) in mean time of follow-up 2. The mean preoperative Mental Health Summary (MHS) gauging the mental state of the total cohort was 41 (29–55), dropping to 39 (25–58) in mean time of follow-up 1 before rising to 50 (35–61) in mean time of follow-up 2. Improvement was significant only regarding the rise between preoperative MHS and mean time of follow-up 2: p = 0.009 (α = 0.05).

**Figure 5:**
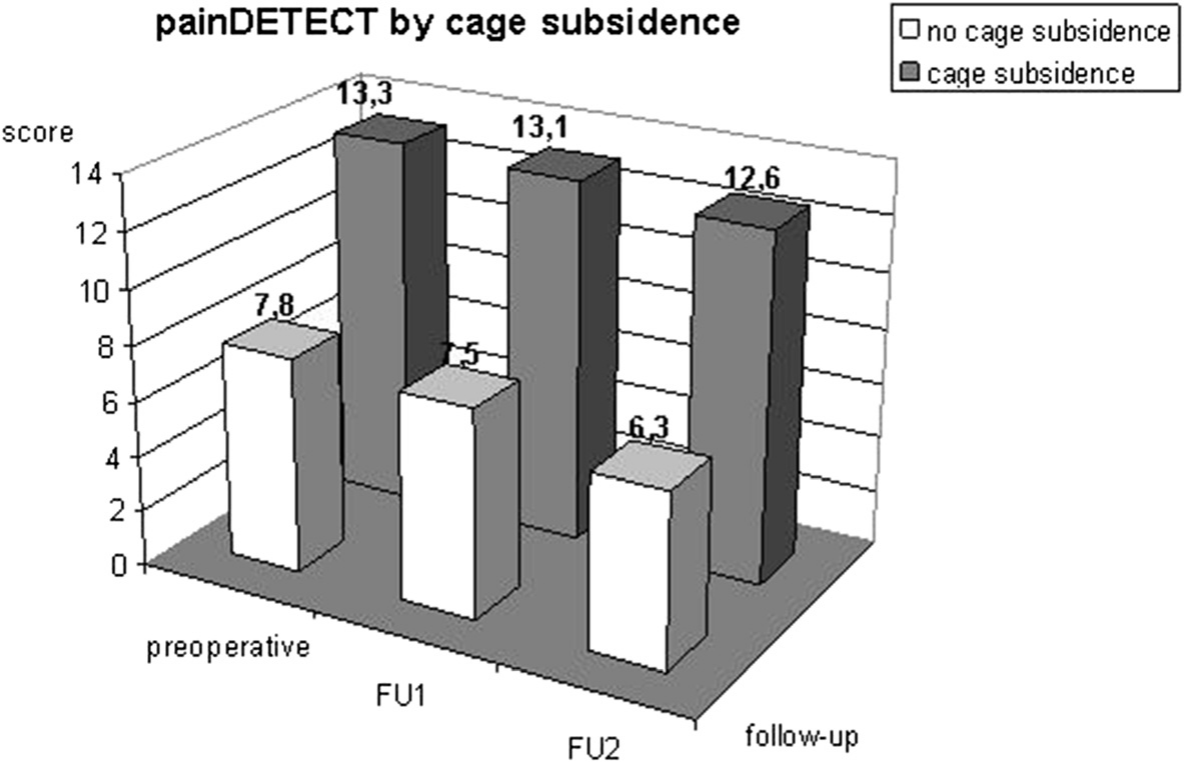
**painDETECT by subsidence: Graph showing the course of pain assessed using the painDETECT score subdivided by cage subsidence; differences between the two groups: p(preoperatively) = 0.03, p (mean time of follow-up 1) = 0.02, p (mean time of follow-up 2) = 0.03 (t-test with a significance level of α = 0.05).**
- The mean preoperative ONDI was 44% (8–78) among patients with subsidence, dropping to 39% (10–72) in mean time of follow-up 1 and 36% (12–64) in mean time of follow-up 2. In patients without subsidence, the mean preoperative ONDI was 59% (22–90), dropping to 39% (32–50) in mean time of follow-up 1 and 36% (24–44) in mean time of follow-up 2. There were no significant differences between the two groups (p = 0.34, α = 0.05).
- The mean preoperative PCS in patients with subsidence was 32 (19–45). This figure rose to 33 (16–50) in mean time of follow-up 1 and 37 (23–49) in mean time of follow-up 2. In the group of patients without subsidence, the preoperative mean of 29 (24–38) increased to 30 (24–38) in mean time of follow-up 1 and 37 (26–47) in mean time of follow-up 2. Here, too, there were no significant differences between the two groups (p = 0.54, α = 0.05).
- Preoperatively, the mean mental quality of life rated using the MHS was 42 (29–55) for the group of patients with subsidence. This dropped to 37 (28–57) in mean time of follow-up 1 before increasing to 48 (35–60) in mean time of follow-up 2. In the group of patients without subsidence, the mean MHS was 38 (31–50), rising to 39 (29–47) in mean time of follow-up 1 and 53 (49–57) in mean time of follow-up 2. Once again, there were no significant differences between the two groups (p = 0.23, α = 0.05).

Below, the results are reported separately for patients with and without subsidence:

- For the group of patients with subsidence, the mean preoperative VAS was 6.3 (3–8), falling to first 4.25 (1–8) in mean time of follow-up 1 and then 3.6 (3–6) in mean time of follow-up 2. In the group without subsidence, the mean preoperative VAS was 5.2 (2–8), which rose to 5.7 (2–9) in mean time of follow-up 1 before dropping again to 3 (2–4) in mean time of follow-up 2. There were no significant differences between the two groups (p = 0.12, α = 0.05).
- The mean preoperative painDETECT score for the group of patients with subsidence was 13.3 (10–21), falling to 13.1 (7–21) in mean time of follow-up 1 and 12.6 (6–20) in mean time of follow-up 2. In the group of patients without subsidence, the mean preoperative score of 7.8 (4–15) dropped to 7.5 (7–8) in mean time of follow-up 1 and then 6.3 (3–10) in mean time of follow-up 2. In each survey period there were significant differences between the two groups: p(preoperative) = 0.03, p(mean time of follow-up 1) = 0.02, p (mean time of follow-up 2) = 0.03 (t-test with a significance level of α = 0.05) (Figure 5).

## Discussion

One aim of surgical treatment is to decompress the neural structures and to restore the height of the intervertebral spaces and the diameter of the intervertebral foramina. In this study, the mean intervertebral spaces increased ventrally by 3.6 mm (1.7–8.5 mm) to 7.5 mm (4.5–10.1 mm) and dorsally from 3.1 mm (mean; 1.2–8.5 mm) to 6.1 mm (mean; 3.9–7.7 mm). This corresponds to an increase in the size of the intervertebral spaces of over 200%. In addition, there was an increase in lordosis in the individual segments from a kyphosis angle of 176° preoperatively to 172.5°. Biederer et al. reported the ventral intervertebral space increasing to 8 mm and the dorsal intervertebral space to 6.9 mm while the dorsal kyphosis angle changed from 177.7° to 175.1° [27], results which are comparable to our own work. In Biederer’s study, the ventral height had decreased from 8 mm to 7.1 mm by the control after 7 months while the dorsal height had dropped from 6.9 mm to 6.3 mm; the kyphosis angle had increased significantly from 175.1° to 176.6°. In our study, too, a decrease was observed in the ventral intervertebral gap from 7.5 mm to 6.2 mm and in the dorsal intervertebral gap from 6.1 mm to 5.4 mm after eight months. After an average of twenty-six months, a further reduction to 5.6 mm ventrally and 4.9 mm dorsally was observed. After eight months, the average angle of the dorsal edge of the vertebrae of 176.9° almost reached the preoperative level. However, this had not decreased any further by 26 months. This loss of height was caused by the cages subsiding into the endplates of the adjacent vertebral bodies. In our work, subsidence was defined as at least 2 mm, as a smaller amount cannot be reliably distinguished from projection artefacts on the lateral X-rays produced [21,27,28]. Whether cage subsidence has a negative impact on the postoperative outcome is controversially discussed in the literature. In our study, only the painDETECT questionnaire revealed a difference between patients with and without subsidence; no differences regarding quality of life, everyday impairment or pain history were indicated by the other scores. Hence, there was only a discrepancy in the assessment of pain between the painDETECT score and the VAS. Strikingly, the preoperative baseline of the painDETECT score (13.3) was almost twice as high as in patients without subsidence (7.8). Both groups declined by about one point to 12.6 and 6.3 respectively by mean time of follow-up 2. This indicates that although there isn’t a difference in tendency between the two groups, they had a different baseline. Although this phenomenon cannot be unambiguously clarified, it appears to be due to the limited number of patients.

Ultimately, however, this work shows that there is no difference in outcome between patients with and without subsidence. This is confirmed by the majority of studies published in recent years. Table 2 lists 18 studies of subsidence following ventral spinal fusion involving a total of 1468 patients published between 1999 and 2013. Hardly any of the papers found outcome to be affected by subsidence. In three studies, no subsidence was observed [22,35,36]. Solely Hahn et al. 2005 found in a study of 80 patients with isolated titanium or carbon fibre cage fusion the outcome to be negatively impacted after three months in patients with subsidence. Then again, the authors noted that subsidence was not thought to be the only reason for the bad outcome [23].

**Table 2:** **Overview of publications addressing subsidence after ventral spinal fusion and how/whether they affect the outcome (number of patients, follow-up in months, subsidence as a percentage, and the impact on outcome)**

**Author/year**	**No. of patients**	**Follow up in months**	**Subsidence in ** **%**	**Impact on outcome**
**Assietti 2002** **[** 20 **]**	24	12	10.5	No
**Barsa 2007** **[** 37 **]**	100	24	45	No
**Bartels 2006** **[** 38 **]**	69	2	29.2	No
**Biederer 1999** **[** 27 **]**	37	6	24	No
**Cabarja 2012** **[** 21 **]**	86	28	17.3	No
**Coric 2013** **[** 22 **]**	74	72	0	
**Gercek 2003** **[** 39 **]**	8	15	55	No
**Hahn 2005** **[** 23 **]**	80	3	30	Negative
**Hwang 2005** **[** 40 **]**	78	11	3.8	No
**Lemcke 2007** **[** 41 **]**	296	12	20.9	No
**Lin 2003** **[** 42 **]**	34	26	9	No
**Mastronardi 2006** **[** 35 **]**	36	12	0	
**Meier 2004** **[** 43 **]**	267	12	18	No
**Moreland 2004** **[** 44 **]**	37	6	22	No
**Pechlivanis 2011** **[** 24 **]**	52	16	19	No
**Salamè 2002** **[** 36 **]**	100	12	0	
**Schmiederer 2006** **[** 45 **]**	54	24	45	No
**Zevgarides 2002** **[** 28 **]**	36	12	33	No

Furthermore, bony fusion does not appear to be impaired by the subsidence of the cage into the vertebral body. In our work, the X-rays indicated successful fusion in all patients and all segments without the formation of pseudarthrosis, regardless of the cage’s subsidence. According to a study by Schmiederer et al., the follow-up after two years of 54 patients after ACDF using a cervical cage indicated stable fusion without pseudarthrosis in all patients, regardless of subsidence [45]. In their follow-up study of monosegmental ACDF Kwon et al. went so far as to declare that there was no correlation between clinical outcome and radiological findings [46].

One reason for subsidence appears to be the intraoperative preparation of the adjacent vertebral bodies. In her study, Hwang et al. observed subsidence only in 3.8%, attributing this low amount to the complete preservation of the endplates of the vertebral bodies thanks to limited, careful debridement [40]. The same conclusion was reached by Fürderer et al. in an animal experiment, in which subsidence was compared depending on the degree of debridement of the vertebral endplates [47]. Limited debridement does not appear to lead to an increase in the rate of pseudarthrosis or a lack of fusion. Hwang et al. reported a fusion rate of 91% after twelve months and 95% after twenty-four months.

In the work presented here, the endplates were always debrided, albeit gently, possibly explaining the higher subsidence of 50% of all cervical cages. However, fusion was observed in all segments at mean time of follow-up 2 after an average of twenty-six months. Lim et al. postulated that the bony endplates must be preserved during surgical preparation, especially in patients with poor bone quality [48].

The available different materials and shapes of cervical cages also appear to affect subsidence markedly. Meier et al. stated in her follow-up of 267 ACDF patients who had received one of six different types of cage systems that spacers made out of titanium tended to subside significantly more than other kinds of implants. It has been suggested that harder materials are more susceptible to subsidence [47,49]. Furthermore, implants with a cubic or a cubic-cylindrical design were found to be less prone to subsidence than planar models. This seems to be attributable to the physiologically different distribution of pressure in the area of the inferior and superior endplates [47].

### Limitations

The main limitations of the study lie in the retrospective study design and the lack of a control group. Additionally, the number of examined patients (17) is far too low to make general valid statements. In particular the number of patients is to low to predict the role of radiographic subsidence depending on number of fused segments and segment high.

## Conclusion

Radiographic subsidence occurred in 50% of the segments treated. This study as well as the literature does not detect any impact of radiographic subsidence on the clinical results. The stand-alone treatment of degenerative cervical spine pathologies is a save method with a high success rate.

### Consent

All patients were informed verbal and in written form and confirmed their approval on a consent form.
